# MERS transmission and risk factors: a systematic review

**DOI:** 10.1186/s12889-018-5484-8

**Published:** 2018-05-02

**Authors:** Ji-Eun Park, Soyoung Jung, Aeran Kim, Ji-Eun Park

**Affiliations:** 1 Research Center for Korean Medicine Policy, Korea Institute of Oriental Medicine, Daejeon, Republic of Korea; 2 Clinical Research Division, Korea Institute of Oriental Medicine, Daejeon, Republic of Korea; 3 Herbal Medicine Research Division, Korea Institute of Oriental Medicine, Daejeon, Republic of Korea; 4 Center for Convergent Research of Emerging Virus Infection, Korea Research Institute of Chemical Technology, Daejeon, Republic of Korea

**Keywords:** MERS, Middle East respiratory syndrome, Infectivity, Severity, Mortality

## Abstract

**Background:**

Since Middle East respiratory syndrome (MERS) infection was first reported in 2012, many studies have analysed its transmissibility and severity. However, the methodology and results of these studies have varied, and there has been no systematic review of MERS. This study reviews the characteristics and associated risk factors of MERS.

**Method:**

We searched international (PubMed, ScienceDirect, Cochrane) and Korean databases (DBpia, KISS) for English- or Korean-language articles using the terms “MERS” and “Middle East respiratory syndrome”. Only human studies with > 20 participants were analysed to exclude studies with low representation. Epidemiologic studies with information on transmissibility and severity of MERS as well as studies containing MERS risk factors were included.

**Result:**

A total of 59 studies were included. Most studies from Saudi Arabia reported higher mortality (22–69.2%) than those from South Korea (20.4%). While the R_0_ value in Saudi Arabia was < 1 in all but one study, in South Korea, the R_0_ value was 2.5–8.09 in the early stage and decreased to < 1 in the later stage. The incubation period was 4.5–5.2 days in Saudi Arabia and 6–7.8 days in South Korea. Duration from onset was 4–10 days to confirmation, 2.9–5.3 days to hospitalization, 11–17 days to death, and 14–20 days to discharge. Older age and concomitant disease were the most common factors related to MERS infection, severity, and mortality.

**Conclusion:**

The transmissibility and severity of MERS differed by outbreak region and patient characteristics. Further studies assessing the risk of MERS should consider these factors.

## Background

Middle East respiratory syndrome (MERS) was first reported in 2012 in Saudi Arabia [1]. Although most patients are linked to the Arabian Peninsula geographically, MERS has been detected in many other parts of the world [2]. A large MERS cluster was also observed in 2015 in South Korea [3].

MERS causes sporadic infection and intrafamilial and healthcare-associated infection. Its symptoms can vary from asymptomatic infection to death. Despite the infection’s association with high mortality, specified antiviral therapy is lacking, especially for patients with concomitant diseases [2].

Many previous studies have assessed the risks of MERS, such as factors dictating severity or an infection risk, yet the indices they present vary. For example, the case fatality rate was found to be 25.9% in the Middle East area, but 20.4% in South Korea [4]. The incubation period was reported to be 6.83–7 days in South Korea [4, 5], but 5.5 in a study using data from multiple areas [6] and 5.2 in Saudi Arabia [7]. Accurate assessment of the risk of MERS is essential for predicting and preventing infection.

A systematic review of the risk of MERS, as covered in previous studies, is potentially helpful for predicting this spread, and its future impact. This study aimed at reviewing the risk of MERS, focusing on indices related to infectivity and severity.

## Methods

We searched international (PubMed, ScienceDirect, Cochrane) and Korean databases (DBpia, KISS) using the term “MERS” or “Middle East respiratory syndrome”, encompassing articles published after 2000. The search process was conducted in October 2017. We also manually searched the reference lists of the included studies.

Human studies were included, while animal studies and reviews were excluded. Only articles in English or Korean were included. Even if a study collected data on humans, such as collecting specimens from religious pilgrims, it was excluded if there were no MERS patients in the study sample. Additionally, case studies including fewer than 20 MERS patients were excluded as they were considered as having insufficient MERS patient numbers and representative information.

The included studies were classified as epidemiologic studies and those covering risk factors of MERS. In the epidemiologic category, indices related to the risk of MERS were divided into two categories; related to infectivity and related to severity. The index related to infectivity included the reproduction number (R), attack rate, incubation period, serial interval, and days from onset to confirmation. The index related to severity included the case fatality rate (CFR), days from onset to hospitalization, days from onset to discharge, days from onset to death, and days from hospitalization to death.

In the risk factor category, factors related to infection, transmission, severity, and mortality of MERS were analysed. Even if the included studies investigated factors that were related to mortality, when they did not analyse risk factors of severity or mortality using appropriate statistical methods (e.g., regression analysis, Cox proportional hazards model) or only compared prevalence factors, we excluded them from the risk factor category. In all categories, we extracted the study period, number of participants, and geographical region where the data were collected using a data extraction form confirmed after pilot assessment.

## Results

**Fig. 1:**
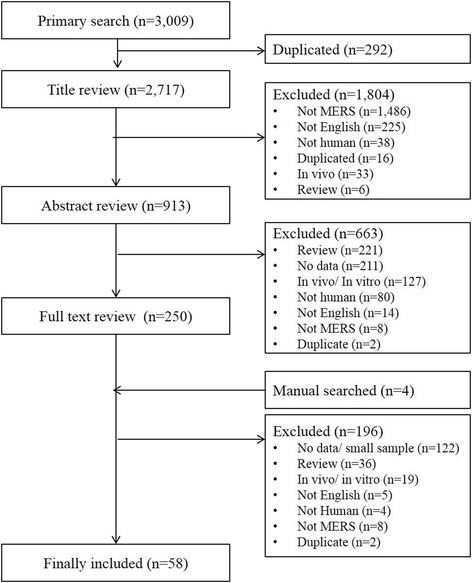
Flow of the systematic review in this study

A total of 3009 studies were searched, and 2717 were reviewed, excluding 292 duplicate studies. After the title and abstract review, a further 1804 and 663 were excluded, respectively. Another four studies were included via a manual search, which left a total of 58 studies for analysis (Fig. 1).

### Epidemiologic studies

The 38 of total 58 included studies were classified as epidemiologic studies (Table 1).

**Table 1:** Epidemiologic studies of MERS, 2012–2017

Author (year)	Country	Study period	No. of patients (M/F) Age of patients	Fatality rate	Contact/ comorbidity	Index related to infectivity	Index related to severity
Ahmed (2017) [31]	Saudi Arabia	2015–2017	537 (370/167) 55 ± 17.9	218/537 (40.6%)	• Comorbidity: 73.9% • Contacts - Hospital-acquired: 38.7% - Household: 9.9% - Camel: 25.3% - Unknown: 26.1%		• Onset to confirmation: 4 days (IQR: 2–7 days)
Alenazi (2017) [12]	Saudi Arabia	2015	130 (66/64) 63.5 (community-acquired), 64.7 (healthcare-acquired), 40.1 (HCW)	51/130 (39.2%)	• Contacts - Community-acquired: 20% - Healthcare-acquired: 46.9% - HCWs: 33.1%	• R - Hospital-acquired: 0.98 for 2nd, 0.64 for 3rd, 0.23 for 4th generation	
El- Bushra (2017) [21]	Saudi Arabia	2015	87 n.r.	n.r.	• Contacts - 20 primary, 39 first, 18 s, 7 third, 3 fourth generation	• Secondary attack rate/10,000: 42 (95% CI: 33–54)	
Kim (2017)	South Korea	2015	186 (111/75) 55	38/186 (19.9%)	• Contacts - Hospital (99.4%) - Household (0.6%) - Community (0%) • Comorbidity - 29/38 fatalities had underlying disease		
Park (2017) [23]	South Korea	2015	25 (13/12) 71^a^ (IQR: 38–86)	25/11 (44.0%)		• Attack rate: 3.7% • Incubation period: 6.1 days	
Sha (2017) [29]	Middle East area/ South Korea	2012–2016	683 (423/260) 50–60 (fatal), 38–46 (non-fatal)	182 (26.6%) (Middle East: 25.9%, South Korea: 13.8%)	• Comorbidity - 67.9% in fatal, 22.2% in nonfatal cases	• Incubation period - 4.5–5 days in Middle East area - 6 days in South Korea	• Onset to confirmation - 8 days in fatal, 4 days in nonfatal in Middle East area - 4 days in fatal, 5 days nonfatal in South Korea • Onset to death - 11.5 days in Middle East area - 11 days in South Korea • Onset to discharge - 14 days in Middle East area - 17 days in South Korea
Sherbini (2017) [32]	Saudi Arabia	2014	29 (20/9) 45 ± 12	10 (34%)	• Comorbidities - Diabetes (31%) - Chronic kidney disease (27%)		• Symptoms to hospitalization: 2.9–5 days
Assiri (2016) [36]	Saudi Arabia	2014–2015	38 (28/10) 51 (range 17–84)	21/38 (55.3%)	• Contacts - 13 HCWs - 15 were associated with 1 dialysis unit		• Onset to death/discharge: 17 days (range 1.0–84.0)
Cho (2016) [5]	South Korea	2015	82 (53/29) 57 (patients and visitors), 38 (HCW)	n.r.	• Contacts - Patients: 40.2% - Visitors: 50% - HCWs: 9.8% • Comorbidity - 24 (29%) had underlying disease	• Incubation period: 7 days (range: 2–17, IQR: 5–10) • Overall attack rate - Patients in the emergency room: 4% (30/ 675) - Visitors: 6% (38/683)	
Halim (2016) [33]	Saudi Arabia	2016	32 (20/12) 43.99 ± 13.03	14/32 (43.8%)			• From symptom to hospitalization - 5.3 ± 3.3 days • Total length of stay - 15 ± 3.6 days
Liu (2016)	Taiwan	2012–2015	1368 (883/476)^a^ 49 (range: 2–90)	35.6% (487/1368) • CFR 7.03% for HCW - 36.96% for non-HCW	• Contacts - Patients: 46.2% - Family members or visitors: 34.9% - HCW: 18.9%		• Onset to death - 13 (4–17) days for HCP - 12 (1–52) days for non-HCW • Onset to confirmation - 6 (1–14) days for HCP - 10 (1–21) days for non-HCW
Mohd (2016) [40]	Saudi Arabia	2014–2015	80 (48/32) 40^a^	• 10% (8/80)	• Comorbidity - Not different from non-MERS groups		
Park (2016) [24]	South Korea	2015	23 (13/10) 66^a^ (range: 31–87) in hospital A; 74.5^a^ (range: 60–82) in hospital B	11/23 (47.8%)	• Generation - 23 in 2nd; 3 in 3rd	• Incubation period - 7.8 days (95% CI: 6.0–10.0) • Serial interval - 14.6 days (95% CI, 12.9–16.5) • Secondary attack rate - 15.8% in hospital A - 14.3% in hospital B	• Time to death^c^ - 12.5 days (IQR: 5.5–19) in hospital A - 11 days (IQR: 9–16) in hospital B
Virlogeux (2016) [25]	South Korea	2015	170 (98/72) 54.6 ± 16.2	36/170 (21%)		Incubation period: 6.9 days (95% CI: 6.3–7.5)	
Chowell (2015) [8]	Saudi Arabia, South Korea	2013–2015	973 MERS and 7634 SARS cases n.r.	n.r.	• Contacts - 43.5–100% were linked to healthcare setting in MERS	R_0_ (95% CI) - MERS: 0.91 (0.36–1.44) - SARS: 0.95 (0.67–1.23) Infection rate of disease among HCWs: - MERS: 13.4–13.5% - SARS: 18.8–57.1%	
Cowling (2015) [26]	South Korea	2015	166 (101/65) 55.4 (range: 16–87)	24/166 (14.5%)	• Contacts - 119 cases had contact with a confirmed case - 30/166 (18%) were healthcare personnel	• Incubation period: 6.7 days • Serial interval: 12.6 days.	
KCDC (2015) [4]	South Korea	2015	186 (111/75) 55^a^ (IQR: 42–66)	36/186 (19.4%)	• Contacts - 44.1% patients exposed in hospitals - 32.8% caregivers - 13.4% HCWs • Comorbidities - 45.2%	• Incubation period - 6.83 days (95% CI: 6.31,7.36)	
Ki (2015) [3]	South Korea	2015	186 (111/75) 50s in men and 60s in women	36/186 (19.4%)	• Generation - 28 in 1st (15.1%); 125 in 2nd (67.2%); 32 in 3rd (17.2%); 2 were not certain • Contacts - Same hospital: 82 (44%) - Family/healthcare aides/visitors: 71 (38%) - HCWs: 31 (17%) • Comorbidities - 77 (41%) had underlying diseases	• Incubation period: 6.5 days (2–16 days).	• From symptom onset to confirmation: 5 days (0–17 days) • From symptom onset to discharge from the hospital: 20 days (8–41 days). • From symptom onset to death: 13 days (1–41 days)
Ministry of Health, South Korea (2016) [27]	South Korea	2015	186 (111/75) 50s (21.6%), 60s (19.9%)	38/186 (20.4%)	• Generation - 28 in 2nd, 120 in 3rd, 26 in 4th, 11 in unclear/ unknown • Contacts - 82 patients in hospital - 63 family members/ visitors - 39 HCWs - 2 others	• Incubation period - 6.83 days (95% CI: 6.31–7.36)	
Noorwali (2015) [37]	Saudi Arabia	2014	261 (171/90) n.r.	110/261 (42%)	• Contacts - 84 HCWs - 177 non-HCWs		
Park (2015) [28]	South Korea	2015	37 (21/16) 51.7 (range: 24–79)	6/37 (16.2%)	• Generation - 1 in 1st, 25 in 2nd, 11 in 3rd • Contacts - 20 patients - 12 relatives of patients - 3 HCWs - 1 unrelated visitors • Comorbidities - 5/6 in fatal, 3/31 in nonfatal	Incubation period - 6 days (95% CI: 4–7 days)	• Symptom onset to confirmation - 6.5 days (95% CI: 4–9) for all cases - 9 days for second cases - 4 days for third cases
Drosten (2014) [22]	Saudi Arabia	2013	26 (17/9) 55^a^ (range: 2–83)	18/26 (69.2%)	• Contacts - 280 household contacts	• Secondary transmission: 4%	
Assiri (2013) [7]	Saudi Arabia	2012–2013	47 (36/11) n.r.	28/47 (60%)	• Comorbidities - 45 had underlying comorbid medical disorders (96%)	• Incubation period: 5.2 days	
Breban (2013) [9]	Multiple areas	2012–2013	64 (44/17) ^b^ 56^a^ (IQR 41–68.5)	38/64 (59.4%)	n.r.	• R_0_: 0.69 (95% CI 0.50–0.92)	
Oboho (2012) [38]	Saudi Arabia	2014	255 (174/81) 45^a^ (IQR:30–59)	93 (36.5%)	• Contacts - 40 of 191 symptomatic were HCWs (20.9%)		n.r.
Penttinen (2013) [41]	Multiple areas	2012–2013	133 (51/77) ^b^ n.r.	45%	• Contacts - 14 primary cases, 129 cases on transmission		• Proportion to ICU: 60 cases (45%)
Estimating the index of infectivity and severity using secondary data							
Author (year)	Country	Study period	Indexes related to infectivity			Index related to severity	
Chang (2017) [18]	South Korea	2015	• R_0_ - 8.097				
Choi (2017) [15]	South Korea, Saudi Arabia	2015	• R_0_ - 3.9 in South Korea - 1.9–3.9 in Saudi Arabia (1.9 in Riyadh, 3.9 in Jeddah)				
Eifan (2017) [13]	Saudi Arabia	2013–2015	• R - 0.85–0.97				
Zhang (2017) [17]	South Korea	2015	• R - 2.5–7.2				
Kim (2016) [16]	South Korea	2015	• R_0_ - 5.4 (95% CI: 4.61–6.19) in period 1 - 0.14 (95% CI: 0.04–0.26) in period 2 • Infectivity of hospitalized patients - 22 (95% CI: 18.73–25.27) in period 1 - 1 (95% CI: 0.16–1.84) in period 2				
Lessler (2016) [39]	Saudi Arabia	2012–2014				• CFR: 22% (95% CI: 18, 25)	
Kucharski (2015) [10]	Multiple areas	2012–2013	• R_0_: 0.47 (95% CI: 0.29–0.80)				
Mizumoto (2015) [35]	South Korea	2015				• CFR: 20.0% (95% CI): 14.6, 26.2).	
Xia (2015) [19]	South Korea	2015	• R_0_ - 4.422 in early stage - 0.385 with control			from hospitalization to death: 15.16 (0–42) (mean, range)	
Cauche-mez (2014) [6]	Multiple areas	2012–2013	• Incubation period - 5.5 (95% CI: 3.6–10·2) • R_0_: 0.8–1.3			• CFR - 74% (95% CI: 49–91) for first cases - 21% (95% CI: 7–42) for second cases	
Chowell (2014) [11]	Saudi Arabia	2013	• R _overall_ - 0.45 (95% CI: 0.29, 0.61) under the surveillance-bias scenario - 0.88 (95% CI: 0.58, 1.20) under the differential-transmissibility scenario				
Poletto (2014) [14]	Middle East area	2012–2013	• R: 0.50 (95% CI: 0.30–0.77)				

*CI* confidence interval, *ICU* intensive care unit, *IQR* interquartile range, *HCW* healthcare worker, *SARS* severe acute respiratory syndrome

^a^Median age, the others are mean age

^b^Information of several participants was missing

^c^Definition is not clear in this study

#### R value

R value, representing the reproduction number, indicates the average number of secondary cases generated by infectious individuals. Thirteen studies reported R value of MERS. Four studies that used data from multiple areas had *R* < 1.0 [6, 8–10]. Studies using Saudi Arabia or Middle East area data reported *R* < 1, at 0.45–0.98 [11–14], though one reported 1.9–3.9 [15]. Studies using South Korea data showed higher values, at 2.5–8.09 [16–19], in the early stage, and < 1 in the later period [20] or with control intervention [19].

#### Attack rate

A total of eight studies reported the attack rate. Four reported the overall or secondary attack rate, and the other four reported the attack rate of specific participant groups. Two studies conducted in Saudi Arabia showed 0.42% [21] and 4% [22] secondary attack rates. Studies in South Korea showed secondary attack rates of 3.7% in one study [23] and 14.3–15.8% in another [24].

Two studies reported the attack rate among healthcare workers (HCWs). One study in South Korea reported a MERS incidence of 1.5% among HCWs [20], and another study using multiple area data reported a 13.4–13.5% infection rate among HCWs [8]. The attack rate among hospital patients was 4% in one study [5] and 22% in the early and 1% in the later period in another [16].

#### Incubation period

The incubation period is the period between infection and appearance of signs of a disease. A total of 12 studies reported the incubation period of MERS. Nine used data from South Korea and showed a 6–7.8 day incubation period [3–5, 23–28]. One study using data from Saudi Arabia reported a 5.2 day incubation period [7], and another using data from multiple areas reported a 5.5 day incubation period [6]. Sha et al. compared the incubation periods between the Middle East area and South Korea and reported 4.5–5 and 6 days, respectively [29].

#### Serial interval

The serial interval of an infectious disease represents the duration between symptom onset of a primary case and of its secondary cases. Two studies used South Korea data, reporting serial intervals of MERS of 12.6 and 14.6 days, respectively [24, 26].

#### Days from onset to confirmation

Among five studies reporting days from onset to confirmation, three studies used data from South Korea. One study analysing all South Korea cases reported 5 days from onset to confirmation [3]. Park et al. reported 6.5 days for all cases, 9 for second generation and 4 for third generation [28]. One study from Taiwan reported 6 days for HCWs and 10 for non-HCWs [30]. A study from Saudi Arabia reported 4 days from onset to confirmation [31]. Sha et al. compared the data from Middle East and South Korea areas and reported 4–8 and 4–5 days, respectively [29].

#### Days from onset to hospitalization

Two studies from Saudi Arabia reported days from onset to hospitalization. One reported 2.9–5 days [32], and the other reported 5.3 days [33].

#### Mortality

Twenty-six studies reported on MERS-related mortality. Ten reported the mortality rate in South Korea as 14.5–47.8% [3, 4, 23–26, 28, 29, 34, 35]; one of which, including all MERS patients in South Korea, reported a mortality rate of 20.4% [27]. Ten studies analysing data from Saudi Arabia reported higher mortality rates, of 22–69.2% [7, 12, 22, 31–33, 36–39], although others reported mortality rates 10% [40] and 19.9% [21]. A Taiwanese study reported a mortality rate of 35.6% [30]. Studies using data from multiple areas reported mortality rates ranging from 26.6% [29] to 59.4% [9, 41].

#### Days from onset to discharge

Three studies reported days from MERS onset to discharge. Sha et al. reported 14 days in the Middle East area and 17 in South Korea [29]. One study from Saudi Arabia reported 17 days [36], and another in South Korea reported 20 [3].

#### Days from onset to death

Two Korean studies reported similar periods of 11–13 days from onset to death: 11–12.5 in Park et al. [24] and 13 in Ki et al. [3]. Although one study from Saudi Arabia reported longer than 17 days from onset to death [36], Sha et al., comparing data between the Middle East and South Korea, reported similar periods of 11.5 and 11 days, respectively [29]. One Taiwanese study also reported a similar period of 12–13 days [30].

#### Days from hospitalization to death

Two studies reported a similar length of hospitalization: 15 [33] and 15.2 days [19].

### Risk factors related to mortality

Of the 20 studies included in the risk factor category, four were duplicates of studies in the epidemiologic category as they had information regarding the epidemiologic index and risk factors (Table 2).

**Table 2:** Factors related to infection, transmission, severity, and mortality of MERS

Author (year)	Study period	No. of participants (Total/death)	Country	Predictors	Significant factors
*Risk factors of infection*					
Alraddadi (2016) [42]	2014	146 (30 cases, 116 controls)	Saudi Arabia	Travel history, animal-related exposure, food exposure, underlying health conditions and behaviors	Direct dromedary exposure in 2 weeks, concomitant with diabetes or heart disease, currently smoking tobacco
Hastings (2016) [43]	2014	78	Saudi Arabia	Nationality, sex, age group, hospital setting, outbreak week	Older age, outbreak week, nationality
*Risk factors of transmission (spreader)*					
Kang (2017) [44]	2015	186	South Korea	Age, sex, comorbidity, symptoms, laboratory test, clinical outcome, phase in transmission, incubation period, symptom onset to isolation, non-isolated in-hospital days, symptom onset to diagnosis	Fever, chest X-ray abnormality in > 3 lung zones, more non-isolated in-hospital days
Kim (2017) [34]	2015	186	South Korea	Underlying respiratory disease, cycle threshold value, symptom onset to diagnosis, no. of contacts, hospitalization or emergency room before isolation	Lower cycle threshold value, hospitalization or emergency room visit before isolation
Majumder (2017) [45]	2015	186	South Korea	Sex, age, comorbidity, case class (HCW, visitor, patient), case outcome (recovered/deceased)	Deceased case outcome
*Risk factors of severity*					
Zhao (2017) [46]	2014–2015	21	Saudi Arabia	CD4 T cell, CD8 T cell, PRNT_50_	Higher PRNT_50_, higher CD4 T cell response
Ko (2016) [48]	2015	45	South Korea	Demographics (age, sex, BMI, underlying disease), symptoms (fever, myalgia, cough, sputum, diarrhea), laboratory test (white blood cell, hemoglobin, thrombocytopenia, lymphopenia, albumin, bilirubin, aspartate transaminase, alanine transaminase, blood urea nitrogen, creatinine, C-reactive protein, lactate dehydrogenase, threshold cycle value of PCR)	• Pneumonia development: older age, fever, thrombocytopenia, lymphopenia, C-reactive protein ≥2 mg/dL, lower threshold cycle value of PCR < 28 • Respiratory failure: male, hypertension, low albumin concentration, thrombocytopenia, lymphopenia, C-reactive protein
Feikin (2015) [47]	2014	102	Saudi Arabia	Age, sex, underlying illness, week of specimen collection, MERS-CoV virus load	• Severity: older age, underlying illness, high MERS-CoV virus load • Mortality: older age, underlying illness, high MERS-CoV virus load
Saad (2014) [49]	2012–2014	70	Saudi Arabia	Age, gender, occupation, acquisition of infection, comorbidity, radiological findings, concomitant infections, laboratory abnormalities	• ICU care: concomitant infection, decreased albumin • Mortality: older age
*Risk factors of mortality*					
Adegboye (2017) [50]	2012–2015	959/317 (33%)	Saudi Arabia	Sex, age, comorbidity, animal contact, camel contact, HCW, secondary contact, clinical experience	Older age, comorbidity, non-HCW, fatal clinical experience
Ahmed (2017) [51]	2014–2016	660/197 (29.8%)	Saudi Arabia	Age, sex, nationality, symptomatic, HCW, severity, source of infection, regions	• 3-day mortality: older age, non-HCW, hospital-acquired infection • 30-day mortality: older age, non-HCW, pre-existing illness, severity, hospital-acquired infection
Sha (2017) [29]	2012–2016	216/56 (25.9%) in Middle East area, 174/24 (13.8%) in South Korea	Middle East Area/South Korea	Age, sex, exposure to camel or other animals, comorbidity, disease progress (days)	Older age (Middle East, South Korea), high comorbidity (Middle East, South Korea), longer days from onset to confirmation of infection (Middle East), longer hospitalized days (Middle East)
Sherbini (2017) [32]	2014	29/10 (34.5%)	Saudi Arabia	Sex, symptoms, history of chronic disease, duration of disease before hospitalization, vital signs, temperature, blood pressure	Older age, gastrointestinal symptoms, longer duration of symptoms prior to hospitalization, diabetes mellitus, chronic kidney disease, smokers, lower blood pressure
Nam (2017) [56]	2015	25/11 (44%)	South Korea	- Epidemiologic (age, sex, hospital, inpatient, staying in the same room as the index case, smoking, preexisting pneumonia, chronic lung disease, incubation period) - Clinical symptom - Laboratory examinations	Male, pre-existing pneumonia, smoking history, incubation period of less than 5 days, leukocytosis, abnormal renal function at diagnosis, respiratory symptoms.
Yang (2017) [52]	2012–2016	1743/559 (32.1%)	Multiple area	Age, sex, comorbidity, epidemic period, contact pattern, country	Older age, comorbidity, epidemic later period
Almekhlafi (2016) [57]	2012–2014	31/23 (74.2%)	Saudi Arabia	Age, comorbidity, initial manifestations, procedures (non-invasive ventilation, invasive ventilation, continuous renal replacement therapy), need for vasopressor	Need for vasopressors
Alsahafi (2016) [53]	2012–2015	924/ 425 (46%)	Saudi Arabia	Age, sex, comorbidities, location of acquisition (household, inpatient, HCW)	Older age, cardiac disease, cancer, household patients, HCW
Virlogeux (2016) [25]	2015	170/36 (21%)	South Korea	Age, sex, incubation period	Older age, shorter incubation period
Cha (2015)	2015	30/5 (16.7%)	South Korea	Age, sex, chronic kidney disease, diabetes, hypertension, comorbidity, estimated glomerular filtration rate, mechanical ventilator	None
Majumder (2015) [54]	2015	159/35 (22%)	South Korea	Five potential covariates were analyzed: sex, age, concurrent health condition status, health care worker status, time from onset to diagnosis	Older age, pre-existing concurrent health conditions
KCDC (2015) [4]	2015	186/36 (19.4%)	South Korea	Sex, age, case classification, respiratory disease, diabetes, cardiac disease, chronic kidney disease, malignancy	Older age, underlying respiratory disease
Das (2015) [58]	2014	55/19 (35%)	Saudi Arabia	Age, chest radiographic score, absolute lymphocyte count, no. of comorbidities, congestive heart failure, hypertension, diabetes	Chest radiographic score
Al Ghamdi (2016) [59]	2014	51/19 (37%)	Saudi Arabia	Beta interferon, alpha interferon, hydrocortisone, Ribavirin, APACHE score	APACHE score
Choi (2016) [55]	2016	186/33 (17.7%)	South Korea	Age, sex, HCW, coexisting medical condition, symptoms at admission, vital signs at admission, laboratory abnormalities at admission, treatment	Age ≥ 55 years, occurrence of dyspnea during the disease course, presence of concomitant medical conditions including diabetes or chronic lung disease, systolic blood pressure < 90 mmHg at admission, leukocytosis at admission, use of mechanical ventilation

*APACHE* acute physiologic and chronic health evaluation, *ICD* intensive care unit, *HCW* healthcare worker, *PCR* polymerase chain reaction, *PRNT* plaque reduction neutralization test

#### Factors related to MERS infection

Two studies reported on the risk factors of MERS infection. Alraddadi et al. [42] analysed the effect of non-human contact, including travel history, animal-related exposure, food exposure, health condition, and behaviour and reported direct dromedary exposure, diabetes or heart disease, and smoking as risk factors of MERS infection. Another study reported older age, outbreak week, and nationality as risk factors [43].

#### Factors related to MERS transmission

Three studies analysed factors associated with spreaders. Non-isolated in-hospital days, hospitalization or emergency room visits before isolation, deceased patients, and clinical symptoms, including fever, chest X-ray abnormality in more than three lung zones, and the cycle threshold value, were related to spreaders [34, 44, 45].

#### Factors related to MERS severity

Four studies reported risk factors of MERS severity. The included studies showed that the PRNT_50_ and CD4 T cell response [46] as well as a high MERS virus load [47] were associated with the severity of MERS. Additionally, male sex; older age; concomitant disease, including hypertension; and symptoms, including fever, thrombocytopenia, lymphopenia, and low albumin concentration, were related to MERS severity or secondary disease [47–49].

#### Factors related to MERS mortality

Fifteen studies reported risk factors of mortality in MERS patients. Older age [4, 25, 32, 49–55] and comorbidity [29, 50–52, 54], including diabetes [32, 55], chronic kidney disease [32], respiratory disease [4, 55], pneumonia [56], cardiac disease, and cancer [53], were the most prevalent in the included studies. Male sex was reported as a risk factor in one study [56]. Smoking [32, 56] and location of acquisition [51, 53] were also reported. While one study noted that HCW, as a profession, was associated with mortality [53], non-HCWs were reported to be related to mortality in two other studies [50, 51].

Additionally, a shorter incubation period [25, 56], longer duration of symptoms [32], more days from onset to confirmation [29], later epidemic period [52], and longer hospitalized days [29] were reported as mortality risk factors.

Symptoms at diagnosis, including abnormal renal function [56], respiratory symptoms [56], gastrointestinal symptoms [32], lower blood pressure [32, 55], and leucocytosis [55, 56], were also found to be associated with mortality in MERS patients.

Severity of illness, [50, 51] such as need for vasopressors [57], chest radiographic score [58], health condition [59], use of mechanical ventilation [55], and occurrence of dyspnoea [55] were also found to increase the mortality risk.

#### Epidemiological index of MERS between the Middle East area and South Korea

The characteristics of MERS differ between South Korea and the Middle East area. The R value of MERS was reported to be below 1 in the Middle East area, except in one study [15], but was from 2.5–8.1 in South Korea [15–19]. Although studies using data from the Middle East area reported 0.42–4% secondary attack rates, studies in South Korea reported 4–6% secondary attack rates for patients or hospital visitors [5], and 3.7–15.8% for the overall attack rate [23, 24]. The MERS incubation period was reported to be 4.5–5.2 days in the Middle East area [7, 29], but this period was found to be slightly longer in South Korea [3–5, 23–28].

The severity of MERS also differed between the Middle East area and South Korea. Mortality of MERS patients was found to be 20.4% in South Korea based on a report including all cases [27], but most studies from Saudi Arabia reported higher rates, from 22 to 69.2% [7, 22, 33, 37–39]. Days from onset to confirmation were similar, 4–8 days in the Middle East area [29, 31] and 4–6.5 days in South Korea [3, 28, 29]. Days from onset to discharge were slightly longer in South Korea, 14–17 days in the Middle East area [29, 36] and 17–20 days in South Korea [3, 29] (Table 3).

**Table 3:** Epidemiologic index of MERS between the Middle East area and South Korea

Index	Saudi Arabia/Middle East area	South Korea (Study including all Korean cases)
Mortality	22–69.2% (Two of ten studies reported lower mortality than 20%)	14.5–47.8% (20.4%)
R-value	0.45–0.98 (Only one study reported 1.9–3.9)	• 2.5–8.1 • Less than 1 in later period or with control intervention
Attack rate	0.42–4%	3.7–15.8%
Incubation period	4.5–5.2 days	6–7.8 days (6.83 days)
Serial interval	–	12.6–14.6 days
Days from onset to confirmation	4–8 days	4–6.5 days (5 days)
Days from onset to hospitalization	2.9–5.3 days	–
Days from onset to discharge	14–17 days	17–20 days
Days from onset to death	11.5–17 days	11–13 days

## Discussion

The transmissibility and severity of MERS were different by outbreak countries, especially between the Middle East area and South Korea. The virus, host, and environmental factors may be the causes of the MERS outbreak-related differences between the two regions. From the standpoint of viral factors, there was a mutation of the MERS coronavirus (MERS-CoV) in the South Korea outbreak. Kim et al. [60] reported a point mutation in the receptor-binding domain of the viral spike protein in MERS-CoV, and another study showed that MERS-CoV in South Korea had higher genetic variability and mutation rates [61]. Individual characteristics can also affect MERS transmission. As previous studies showed, there is an association between older age and MERS infection [43], severity [48], and mortality [4, 50], and the population structure may be related to transmission and severity. Additionally, individuals aware of MERS were found to be more likely to practice preventive behaviour [62], which differed by demographic characteristics [63, 64]. The transmission environment may also contribute to the difference. While many MERS cases were contracted through exposure to camels in Saudi Arabia [42], the South Korea outbreak involved multiple generations of secondary infections caused by intra-hospital and hospital-to-hospital transmission [3, 65]. Strategies considering various factors are therefore needed to assess the impact of MERS and to better control its spread.

Although several studies have reported the overall R value [9, 10, 14, 19], others have shown that this value this can be variable based on the generation or a control intervention [11, 16, 19]. Especially in the South Korea epidemic, the R value was particularly high in the early stage or first generation, at 4.42–5.4, though it later decreased to 0.14–0.39 [16, 19]. Further studies should consider and analyse the variation of the R value depending on the period or control intervention.

While earlier studies on infectious diseases assumed a homogeneous infection ability of a population, recent studies have shown the existence of so-called super spreaders, individuals with a high potential to infect others in many infectious diseases, including Ebola and severe acute respiratory syndrome (SARS) [66]. The role of the super spreader is also important in the spread of MERS. In South Korea, 83.2% of MERS patients were associated with five super-spreading events [27]. Stein et al. [67] asserted that super spreaders were related with the host, pathogen, and environmental factors, and Wong et al. [66] reported that individual behaviours could also contribute to disease spread.

There are variations in the mortality and attack rates among studies using South Korea data. For example, Park et al. [24] reported a 47.8% MERS mortality, while reports from the Korean Ministry of Health and Welfare showed 20.4% MERS mortality. This disparity may, in part, be due to small sample sizes. Park et al. [24] included only 23 patients because the study was conducted in an early phase of a MERS outbreak. We excluded studies that included cases with < 20 subjects, which were mostly case series, to reduce those types of biases.

The present review found that older age and concomitant disease were risk factors of MERS infection and mortality. These results are consistent with a recent systematic review that reported older age, male, and an underlying medical condition as predictors of death related to MERS [68]; therefore, these factors should be prioritized in protection and treatment procedures.

One limitation of this study was the possibility of subject duplication. Especially in South Korea, the Korean government publishes MERS reports that include all patients. The epidemiologic index in other studies might be biased since they included partial Korean patients and were analysed in the middle of an outbreak. However, we included those studies because they showed the characteristics of MERS in different situations and different stages.

We did not conduct a meta-analysis because of the small number of studies for each index, which might be another limitation of this study. Although this study reviewed the risk factors of MERS and their impact, assessing the effect size of each risk factor is important. More studies investigating the effect of risk factors on MERS need to be constantly conducted.

## Conclusion

Most studies on the transmissibility and severity of MERS have originated from Saudi Arabia and South Korea. Even though the R_0_ value in South Korea was higher than that in Saudi Arabia, mortality was higher in Saudi Arabia. The most common factors behind MERS infection and mortality were older age and concomitant disease. Future studies should consider the risk of MERS based on the outbreak region and patient characteristics. The results of the present study are valuable for informing further studies and health policy in preparation for MERS outbreaks.

## Abbreviations

CD4: Cluster of differentiation 4
CFR: Case fatality rate
MERS: Middle East respiratory syndrome
PRNT: Plaque reduction neutralization test
SARS: Severe acute respiratory syndrome
